# Immunocytes in the tumor microenvironment: recent updates and interconnections

**DOI:** 10.3389/fimmu.2025.1517959

**Published:** 2025-04-14

**Authors:** Jiyao Yu, Li Fu, Rui Wu, Linyi Che, Guodong Liu, Qinwen Ran, Zhiwei Xia, Xisong Liang, Guanjian Zhao

**Affiliations:** ^1^ Department of Ultrasound, The Second Affiliated Hospital, Chongqing Medical University, Chongqing, China; ^2^ Institute of Psychiatry, Psychology and Neuroscience, King’s College London, London, United Kingdom; ^3^ Department of Neurosurgery, The Second Affiliated Hospital, Chongqing Medical University, Chongqing, China; ^4^ Department of Gastroenterology, The Second Affiliated Hospital, Chongqing Medical University, Chongqing, China; ^5^ Department of Neurosurgery, Jiangyou People’s Hospital, Mianyang, China; ^6^ Department of Neurology, The First Affiliated Hospital of Chongqing Medical University, Chongqing, China; ^7^ General Practice Department, Wufu Town Hospital, Chongqing, China; ^8^ Department of Neurology, Hunan Aerospace Hospital, Hunan Normal University, Changsha, China; ^9^ Department of Neurosurgery, Xiangya Hospital, Central South University, Changsha, China; ^10^ National Clinical Research Center for Geriatric Disorders, Xiangya Hospital, Central South University, Changsha, China

**Keywords:** cancer immunity, immunosuppression, lymphocytes, myeloid cells, immune checkpoints

## Abstract

The tumor microenvironment (TME) is a complex, dynamic ecosystem where tumor cells interact with diverse immune and stromal cell types. This review provides an overview of the TME’s evolving composition, emphasizing its transition from an early pro-inflammatory, immune-promoting state to a later immunosuppressive milieu characterized by metabolic reprogramming and hypoxia. It highlights the dual roles of key immunocytes—including T lymphocytes, natural killer cells, macrophages, dendritic cells, and myeloid-derived suppressor cells—which can either inhibit or support tumor progression based on their phenotypic polarization and local metabolic conditions. The article further elucidates mechanisms of immune cell plasticity, such as the M1/M2 macrophage switch and the balance between effector T cells and regulatory T cells, underscoring their impact on tumor growth and metastasis. Additionally, emerging therapeutic strategies, including checkpoint inhibitors and chimeric antigen receptor (CAR) T and NK cell therapies, as well as approaches targeting metabolic pathways, are discussed as promising avenues to reinvigorate antitumor immunity. By integrating recent molecular insights and clinical advancements, the review underscores the importance of deciphering the interplay between immunocytes and the TME to develop more effective cancer immunotherapies.

## 1 Overview of the tumor microenvironment

### 1.1 Composition and dynamic changes

**Figure 1:**
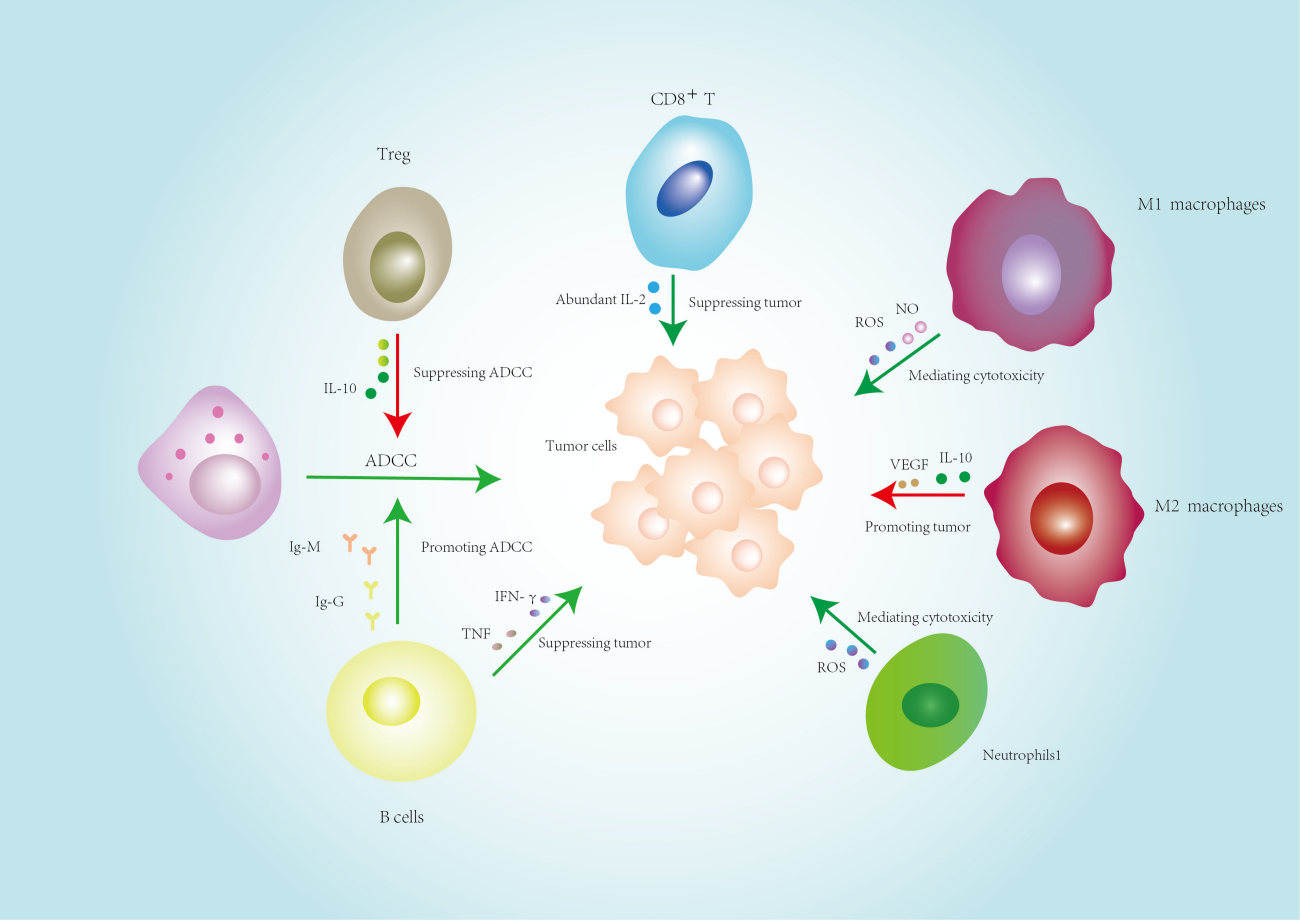
Cross-talk among immune cells in the TME: balancing antitumor and protumor functions.

**Figure 2:**
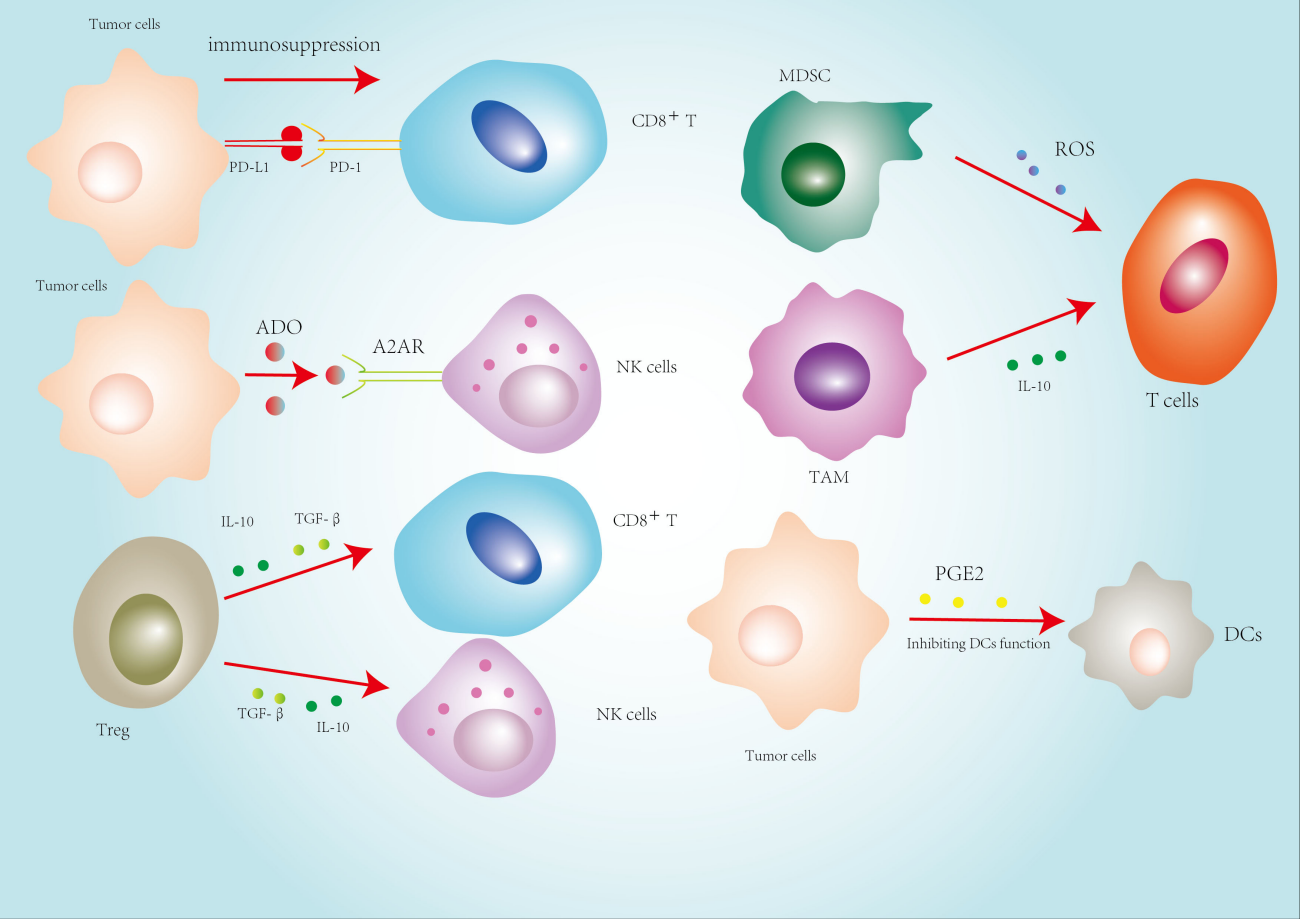
Immunosuppressive network in the tumor microenvironment (TME).

The tumor microenvironment (TME) is composed of diverse cellular populations, including tumor-associated cells, immune cells, and stromal cells, characterized prominently by its dynamic composition, especially during later stages of tumor progression. Within this complex and evolving environment, cross-talk among immune cells is crucial, as it dynamically balances antitumor and protumor functions, ultimately influencing tumor development and metastasis (**Figure 1**). During the early phase of oncogenesis, TME is immune-promoting with a high pro-inflammatory signal, causing immunocytes to display a pro-inflammatory phenotype. As the tumor progresses, the TME gradually transforms into an immunosuppressive environment (**Figure 2**) with low glucose concentration, high fatty acid, lactic acid, adenosine concentrations, low oxygen, low pH, and low amino acid concentration. Moreover, immunocytes tended to show an inhibitory phenotype. All the alterations of TME are governed mainly by neoplastic cells instead of the body itself. All non-tumor cells possess complicated functions in this complex, dynamic, uncontrolled microenvironment. Immune cells within them will differentiate into subgroups with distinct phenotypes, metabolic properties, and processes, which play an antitumor or tumor-promoting function, respectively (1). For instance, at the early stages of oncogenesis, infiltrating macrophages adopt an M1-like phenotype that promotes the destruction of tumor cells and the inhibition of angiogenesis. As the tumor progresses, significant heterogeneity in oxygen content in a tumor mass forms. When macrophages are recruited and entrapped in hypoxic tumor areas, macrophages’ polarization can be altered to an M2-like pro-tumor phenotype (2, 3). In a genetically engineered mouse lung adenocarcinoma model, Tregs, known for their immunosuppressive properties, are observed in tertiary lymphoid structures actively suppressing potent anti-tumor responses (4). As for myeloid-derived suppressor cells (MDSCs)-an immunosuppressive innate cell population (5), overcoming key cellular stress mediators or preventing metabolic polarization of MDSCs in tumors switched MDSCs into cells that prime anti-tumor T cell immunity or directly kill cancer cells (5).

Studying immune cells has enabled the rapid development of new immunotherapies and the identification of clinical biomarkers (6).

### 1.2 Immune cells and tumor-driven remodeling

Immune cells in TME can be categorized into two main groups: innate and adaptive immune cells. Among them, innate immune cells include macrophages, neutrophils, dendritic cells, myeloid-derived suppressor cells, mast cells, and NK cells, while adaptive immune cells include T and B cells. The metabolism of tumor cells influences the secretion of cytokines, chemokines, and growth factors, which in turn affects the development of tumor cells. Among them, innate immune cells can rapidly recognize tumor antigens and phagocytose and deliver relevant tumor antigens. In contrast, adaptive immune cells are activated by exposure to specific antigens and utilize immune memory to regulate immune responses (7). In the early stages of tumorigenesis, the predominant cells in the TME are infiltrating inflammatory cells, bone marrow-derived hematopoietic and endothelial progenitor cells, and cancer-associated fibroblasts (CAF) (8). As the tumor grows, neutrophils and macrophages are recruited into the tumor tissue and release cytokines, pro-inflammatory factors, and reactive oxygen species, which promote the recruitment and proliferation of T cells and NK cells, promoting tumor cell clearance in turn (7). In addition, the progress of cancer causes a gradual decrease in cytotoxic CD8^+^ T cells and NK cells, an increase in dysfunctional CD8^+^ T cells, immunosuppressive CD4^+^ FoxP3^+^ Tregs, and regulatory B cells. Additionally, macrophages become polarized to an M2-like state, and neutrophils become activated due to extracellular matrix changes. This leads to the release of vascular endothelial growth factor (VEGF) and the production of matrix metalloids that stimulate angiogenesis. CD4^+^ T cells are biased towards a pro-inflammatory Th2 phenotype, while dendritic cells (DCs) become defective in maturation and function. The co-evolved immune milieu is profoundly altered, presenting a state of immunosuppression, immune system rejection, neglect, and finally causing tumor metastasis (9). During tumorigenesis and development, immune cells do not simply have anti-tumor or pro-tumor effects but are two-sided and convertible. Antitumor immune cells include T cells, natural killer cells, classically activated macrophages (M1 macrophages), neutrophils 1, B cells, conventional dendritic cells, and protumor cells. In the next section, the immune cells in TME, from anti-tumor immunity and pro-tumor immunity, will be introduced in detail.

## 2 Immune cells in the TME

### 2.1 Antitumor immune cells

#### 2.1.1 T cells

T cells are the main cell types that are antitumors in adaptive immunity (10). The naive T cells are divided into CD8^+^T and CD4^+^T cells. Activated by antigen-presenting cells, CD8^+^T cells can be differentiated into cytotoxic T lymphocytes (CTL) and memory T cells. Also, CD4^+^ naïve T cells differentiate into different subtypes. For instance, Th1, Th2, Th17 and Treg. Furthermore, while CD8^+^ T cells play a central role in direct tumor cell killing, CD4^+^ T cells are critical for orchestrating the overall immune response through cytokine secretion and support of other immune effectors. For example, Th1 subtypes exert antitumor effects by aiding cytotoxic CD8^+^ cells and B cells. At the same time, Th2 subtypes secrete anti-inflammatory mediators with pro-tumorigenic effects (9).

##### 2.1.1.1 CD8^+^T cells

###### 2.1.1.1.1 CD8^+^ T cell–derived cytokines and their dual roles

CD8^+^ T cells produce a variety of cytokines—including interleukin−2 (IL−2), interferon−γ (IFN−γ), and tumor necrosis factor−α (TNF−α)—that play pivotal roles in regulating immune responses. Initially, IL−2 was identified as a T cell growth factor with a strong capacity to promote the expansion of cytotoxic CD8^+^ T cells (11, 12). In this context, IL−2 binds to its receptor (IL−2R) and triggers several downstream signaling pathways. For example, in effector T cells and natural killer (NK) cells, IL−2 binding activates the JAK1–JAK3–STAT5, PI3K–mTOR1, and MAPK pathways via CD122 and CD132, whereas in regulatory T (Treg) cells—where high levels of the PI3K antagonist PTEN are present—the JAK1–JAK3–STAT5 pathway predominates (13). Consequently, IL−2R signaling induces the transcription of genes that support not only the proliferation of effector T cells, NK cells, and ILC2s but also that of Treg cells.

Decades of research have since revealed the dual functionality of IL−2 (14). Specifically, at low doses IL−2 preferentially stimulates Treg cells—which express high−affinity trimeric receptors (IL−2Rαβγ)—thus promoting immunosuppressive effects (15, 16). In contrast, at high doses, once the receptors on Treg cells become saturated, excess IL−2 engages intermediate−affinity receptors (IL−2Rβγ) on effector T cells and NK cells, thereby enhancing immune activation and antitumor responses (17). Indeed, following extensive clinical evaluation, high−dose recombinant IL−2 (aldesleukin, trade name Proleukin) became the first FDA−approved immunotherapy for metastatic melanoma and renal cell carcinoma (18, 19).

###### 2.1.1.1.2 IFN−γ: a double-edged sword

In addition to IL−2, IFN−γ secreted by CD8^+^ T cells plays a crucial role in modulating antitumor immunity. On one hand, IFN−γ binds to its receptor and activates the JAK/STAT pathway, leading to the production and activation of IRF−1 and subsequent modulation of programmed cell death ligand−1 (PD−L1) expression in tumor cells (20, 21). In fact, recent studies have demonstrated that IFN−γ substantially influences PD−L1 expression in the glioma microenvironment, where researchers have even introduced an IFN−γ score to correlate with PD−L1–related gene expression (22). On the other hand, it is important to note that IFN−γ also exhibits immunosuppressive properties (23). For example, it can induce indoleamine−2,3−dioxygenase (IDO) activity in dendritic cells (DCs) (24) and further upregulate PD−L1 on tumor cells (25, 26). Moreover, in settings such as MUC1−C–activated triple−negative breast cancer, the IFN−γ–driven JAK1→STAT1→IRF1 pathway leads to the induction of immunosuppressive effectors like IDO1 and COX2/PTGS2 (27). Additional effects include promoting the accumulation of myeloid−derived suppressor cells (MDSCs) (28) and reducing the responsiveness of T and NK cells to IFN−γ via nitric oxide production (29). Furthermore, gangliosides can act in concert with IFN−γ and PD−L1 to diminish the DC−stimulating potential of CD8^+^ T cells (30, 31). Notably, in experimental glioma models, the antitumor effects of cytokine–antibody conjugates (e.g., L19 antibodies coupled with IL−12 or TNF−α) were completely abrogated when either CD4^+^ or CD8^+^ T cells were depleted (32).

###### 2.1.1.1.3 TNF-α: Multifaceted Roles in Immunity

TNF‑α is primarily produced by macrophages, but also by a variety of other cells, including NK cells, T lymphocytes, smooth muscle cells, fibroblasts, and others (33). Release of TNF‑α occurs in response to inflammatory stimuli and cytokines, including peptidoglycan, lipopolysaccharide, and other bacterial components (34). Two receptors exist for TNF‑α: 1) Tumor necrosis factor receptor 1 (TNFR1), which preferentially binds soluble TNF‑α and is found almost ubiquitously on the surface of cells, and 2) Tumor necrosis factor receptor 2 (TNFR2), which is found on cells of the hematopoietic lineage and has specificity for the transmembrane form of TNF‑α (32). The biological effect of TNF‑α binding to its receptor depends on the receptor activated and the cellular state during activation. Stimulation of TNFR1 activates downstream inflammatory mediators through AP1, MAPK, and NF-kB pathways (35). Conversely, the biological role and downstream effects of TNFR2 stimulation are less well understood.

Conflicting results have been published on the role of TNF in the T‑cell immune response toward cancer cells. On one hand, TNF acts as an effector molecule in CD8⁺ T‑cell–triggered cell death of cancer cells (36) and serves as a costimulatory cytokine able to enhance naive CD8⁺ T‑cell proliferation and cytokine secretion (37, 38). In association with IFN‑γ, TNF induces senescence in cancer cell lines (39). Additionally, TNF is essential for establishing antitumor immune responses by facilitating dendritic cell maturation, CD8⁺ T‑cell activation, and tumor infiltration (40, 41). However, TNF also promotes activation-induced cell death in CD8⁺ T cells (42), potentially limiting the duration of immune responses. Furthermore, TNF may contribute to an increase in regulatory T cells (43, 44), B cells (45), and myeloid-derived suppressor cells (46, 47). In adoptive transfer therapy involving CD8⁺ T lymphocytes in mice, TNF can cause melanoma cells to dedifferentiate. Such dedifferentiation is linked to decreased expression of melanocytic antigens, potentially facilitating tumor relapse (48).

###### 2.1.1.1.4 Metabolic constraints imposed by the tumor microenvironment

In the TME, the Warburg effect leads to the production of lactic acid, which creates an acidic environment that negatively impacts CTL function. Lactic acid impairs CTL chemotaxis and respiratory activity by multiple mechanisms: it reduces the recruitment of CTLs, captures pre-existing T cells, and, once internalized via the SMCT2 transporter by CD4^+^ helper T cells and CTLs, inhibits glycolysis through downregulation of Hexokinase 1 or inhibition of phosphofructokinase (49, 50). Furthermore, lactic acidosis not only impairs the exocytosis of lytic granules but also suppresses cytokine release (including TNF−α, IL−2, and IFN−γ) via effects on MAPK p38 and JNK/c−Jun signaling pathways (51). As CTLs become functionally exhausted in such conditions, their metabolic profile shifts from oxidative phosphorylation (OXPHOS) to glycolysis, leading to decreased glucose uptake, low PGC−1α expression, and ultimately compromised ATP synthesis and effector function (52–57).

###### 2.1.1.1.5 Genomic and single−cell insights into CTL dysfunction

Recent functional genomic studies have further underscored the importance of metabolic regulation in immune evasion. For example, Lawson et al. (2020) performed genome-wide CRISPR screens in diverse mouse cancer cell lines and identified a core set of 182 cancer-intrinsic CTL-evasion genes. Many of these genes are involved in pathways regulating mitochondrial metabolism, autophagy, and interferon signaling – processes that enable tumor cells to survive under metabolic stress and evade CTL-mediated killing. These findings suggest that alterations in mitochondrial translation, the electron transport chain, and autophagy not only support tumor cell fitness in the harsh TME but also indirectly contribute to CTL dysfunction (58).

Further adding to our understanding, recent single−cell CRISPR screens *in vivo* have mapped the gene regulatory networks that govern CTL fate within tumors. This study revealed that transcription factors—including IKAROS, ETS1, and RBPJ—are key determinants of the balance between precursor exhausted T (Tpex) cells and terminally exhausted T (Tex) cells, with their activities tightly linked to mTORC1‐mediated metabolic reprogramming. For instance, perturbation of ETS1 was shown to boost the differentiation of CTLs toward a more effective effector state, whereas RBPJ deficiency promoted the accumulation of Tex cells with enhanced proliferative and cytotoxic features. These insights underscore that the transition from a stem‐like, metabolically quiescent Tpex state to an exhausted Tex state is not only driven by chronic antigen stimulation but also by coordinated changes in cellular metabolism and transcriptional regulation. Such findings imply that therapeutic strategies aimed at modulating both metabolic pathways and gene regulatory networks may synergistically restore CTL functionality (59).

###### 2.1.1.1.6 The role of hypoxia and HIF−1α

HIF−1α is a crucial component of the hypoxia response and is associated with the occurrence and growth of numerous malignant tumors, further inhibiting CTL infiltration by disrupting chemotaxis. Upon internalization of lactic acid via the SMCT2 transporter, both CD4^+^ helper T cells and CTLs exhibit inhibited glycolysis and reduced motility due to either inhibited phosphofructokinase or downregulated Hexokinase 1 (50). It is significant to note that HIF−1α inhibition synergizes with anti−PD−1 therapy to inhibit tumor development. Research findings indicate that suppressing HIF−1α—through pharmacologic or genetic means—can eliminate immune evasion mediated by PD−L1, by reducing its expression on tumor−associated macrophages (TAMs) and malignant cells, which in turn triggers reactivation of tumor−infiltrating lymphocytes (TILs) and leads to tumor rejection (60). In a melanoma model, HIF−1α inhibition was also shown to increase the accumulation of natural killer (NK) cells and CTLs in the tumor bed by upregulating chemokines such as CCL2 and CCL5, thereby enhancing the effectiveness of anti−PD−1 therapy and peptide vaccines (60). Moreover, although anti−PD−1/PD−L1 and anti−CTLA−4 therapies are among the most effective immunotherapies for cancer, they are frequently accompanied by significant immune−related complications. In one study, anti−CTLA−4 therapy combined with the HIF−1α inhibitor echinomycin achieved effects comparable to the combination of anti−CTLA−4 and anti−PD−1 antibodies (60). Furthermore, recent investigations have found that hypoxia selectively up−regulates PD−L1 on myeloid−derived suppressor cells (MDSCs) via HIF−1α binding to a hypoxia−response element (HRE) in the PD−L1 proximal promoter; inhibiting PD−L1 under hypoxic conditions can reverse MDSC−mediated T cell suppression. Therefore, combination therapies that target tumor hypoxia by concurrently inhibiting PD−L1 and HIF−1α may further enhance the immune response against tumors (60–62).

###### 2.1.1.1.7 CTL cytotoxicity and additive effects in solid tumors

In TME, CD8^+^ T cells remain essential for tumor immunity. Beyond the challenges imposed by metabolic reprogramming, emerging evidence has revealed that CTL-mediated cytotoxicity in tumors is not an all-or-nothing process but rather an incremental one. Recent live-cell imaging studies have introduced the concept of “additive cytotoxicity,” in which CTLs deliver multiple sublethal hits that accumulate over time to induce tumor cell death. In solid tumors such as melanoma, individual CTL–target cell interactions often result in perforin-mediated plasma membrane damage, transient nuclear envelope rupture, DNA damage, and the generation of reactive oxygen species (ROS). Although these sublethal events can be repaired by tumor cells through mechanisms involving calcium influx-triggered membrane resealing via the ESCRT machinery and activation of DNA damage repair pathways, repeated CTL contacts can overwhelm the repair capacity and trigger apoptosis. This additive effect is further enhanced by factors such as increased CTL density, prolonged immune synapse stability, and interventions that inhibit tumor cell repair processes. These insights have spurred novel immunotherapeutic approaches that aim to boost CTL efficacy by promoting CTL swarming, stabilizing cytolytic synapses with bispecific antibodies, or combining adoptive cell transfer with agents targeting cellular repair pathways (63).

###### 2.1.1.1.8 Comparative insights: hematological malignancies versus solid tumors

Moreover, recent studies in solid tumors have uncovered additional signaling pathways that critically modulate CTL function. For instance, research by Zhang et al. has demonstrated that within the solid tumor microenvironment, factors such as adenosine and regulatory T cells induce the expression of PARP11 in CTLs. This upregulation promotes ADP ribosylation of β-TrCP, thereby stabilizing β-TrCP and enhancing the ubiquitination and degradation of the type I interferon receptor IFNAR1. The consequent downregulation of IFNAR1 compromises the pro-survival and activation signals mediated by type I interferons, ultimately diminishing CTL viability and cytotoxicity. Importantly, genetic or pharmacologic inhibition of PARP11 (for example, using Rucaparib) has been shown to restore IFNAR1 expression and boost CTL—and even CAR T cell—tumoricidal activity in solid tumors (64).

Notably, similar principles apply to hematological malignancies where CTL function is modulated by distinct immunosuppressive signaling pathways. In this context, key pathways—including the PD-1/PD-L1 axis, CTLA-4-mediated inhibitory signaling, and metabolic regulation via indoleamine 2,3-dioxygenase (IDO)—play crucial roles in restraining CTL activity. For example, leukemia and lymphoma cells can express high levels of PD-L1 and CTLA-4 ligands, thereby dampening CTL responses, while IDO-driven tryptophan depletion further impairs CTL proliferation and cytotoxicity. Moreover, spontaneous CTL responses targeting these immunosuppressive molecules have been observed, highlighting their potential as therapeutic targets in blood cancers (65).

In summary, while CTLs in solid tumors encounter complex challenges—including both physical damage from additive cytotoxicity and biochemical suppression via pathways such as PARP11-mediated IFNAR1 downregulation—the immunosuppressive mechanisms in hematological malignancies are more prominently governed by immune checkpoint pathways and metabolic enzymes. Understanding these distinct yet occasionally overlapping signaling mechanisms is crucial for designing tailored immunotherapeutic strategies that can effectively reinvigorate CTL function across diverse tumor types.

###### 2.1.1.1.9 Therapeutic implications and emerging immunotherapies

These recent insights into the metabolic, transcriptional, and damage-response regulation of CTL function highlight that the balance between energy metabolism, sublethal damage accumulation, and repair activity is a critical determinant of CTL efficacy. Therapeutic strategies aimed at modulating mitochondrial metabolism, autophagy, and key transcriptional regulators, along with approaches that promote additive cytotoxicity, hold promise for reinvigorating CTLs, thereby improving antitumor immunity and enhancing responses to immunotherapies.

Several immunotherapies, such as tumor vaccines, chimeric antigen receptor T cells (CAR-T), and bispecific T-cells, have been developed to specifically target and kill tumor cells (24). Leukemia and lymphoma have been treated using CAR-T in recent years (66, 67). Long-lasting anti-CD19 CAR-T cell characterization, both functional and molecular, is described in depth in a recent work. The anti-leukemia response was shown in two separate phases: a CD8^+^ or CD4^-^CD8^-^Helios^hi γδ CAR-T cell-dominated initial phase, which was followed by a majority proliferative CD4^+^ CAR-T cell population in subsequent years (68). ID3 is crucial for promoting the growth of NK cells from bipotential NK/T progenitor cells in the thymus (69), and SOX4 has been proven to regulate the formation of invariant natural killer T cells by inducing microRNA-181 (Mir181) to boost the TCR signaling pathway (70). Although B7-H3 is expressed in central nervous system malignancies, this treatment has also been gradually applied to the treatment of gliomas in recent years (71–73). B7-H3 CAR-T cells are repeatedly used for local treatment of tumors, with patients showing continuous clinical and imaging improvement within 12 months. Targeted mass spectrometry of CSF biological samples demonstrates regulation of B7-H3 and key immune analytes (CSF-1, CXCL13, CD14, CD163, and VCAM-1) (74). Although dose and other aspects need to be further verified in clinical investigations, it is evident that these treatments have significant potential.

###### 2.1.1.1.10 Expanding the arsenal: the role of invariant NK T cells

In addition, invariant NK T (iNKT) cells, CD1d-restricted lipid-specific T lymphocytes, enable the coordination of innate and acquired immunity and act as tumor immune surveillance. Unlike conventional T cells that recognize peptide antigens presented by polymorphic MHC molecules, iNKT cells respond to lipid antigens bound to the non-polymorphic CD1d molecule, giving them a unique advantage in targeting tumor cells that present alternative or non-peptide antigens. Once activated by glycolipid antigens (e.g., α-galactosylceramide, α-GalCer) on CD1d^+^ antigen-presenting cells, iNKT cells produce large amounts of IFN-γ and IL-4. Simultaneously, they can exert cytotoxic effects against tumor cells via perforin/granzyme and FasL. What’s more, following activation of iNKT cells, high-functioning NK cells can be imprinted to exhibit antitumor effects via co-stimulatory molecule- (NKG2D and DNAM-1) and IFN-γ-dependent mechanisms. In the meantime, DC maturation caused by iNKT cells leads to conversion from tolerogenic to immunogenic adaptive immune responses, boosting downstream tumor-specific CD4^+^ or CD8^+^ T cell responses, and might potentially be exploited in adjuvant approaches for immunotherapy.

Several immunotherapies based on iNKT cells have been developed. To be specific, autologous α-GalCer-pulsed DC therapy, loading DCs with an iNKT cell agonist (such as α-GalCer), can overcome anergy and elicit strong IFN-γ production, invigorating adaptive immune responses, including CD8^+^ cytotoxic T lymphocytes and NK cells. In addition, iNKT cells could be engineered similarly to conventional T cells—yielding CAR-iNKT cells. Early-stage studies in other cancer models (e.g., neuroblastoma) suggest that CAR-iNKT cells may effectively target tumors while retaining the ability to modulate the microenvironment through innate-like pathways. Recently, A research team introduced the concept of artificial adjuvant vector cells (aAVCs) as a potential new type of cancer vaccine platform that leverages *in vivo* iNKT-licensed dendritic cell therapy. These engineered cells display the α-GalCer–CD1d complex externally and incorporate tumor antigens (e.g., OVA, MART-1, Trp2, or WT1) internally. In a murine model of lung metastasis triggered by intravenous B16 melanoma injection, administering aAVCs significantly decreased the number of metastatic foci compared with control treatments. Furthermore, two weeks after MO4 (B16 engineered to express OVA) inoculation, aAVC therapy suppressed the development of sizable cutaneous tumors (75).

##### 2.1.1.2 CD4^+^ T cells

As mentioned above, based on their cytokine environment, CD4^+^ T cells can develop into multiple phenotypically diverse subsets, with Th1 and Th2 cells being the two primary types. Th1 cells are the primary subset of T helper (Th) cells engaged in the anti-tumor immune response. They generate cytokines such as interferon-gamma (IFN-γ), interleukin (IL) − 2, and tumor necrosis factor α (TNFα) (76). On the other hand, Th2 cells support tumor growth and metastasis while inhibiting the immune system (77). More specifically, Th1 cells increase the proliferation of CD8^+^ T cells and upregulate the production of granzyme B, which in turn increases the ability of immune cells to destroy (78). In addition, Th1 cells stimulate DCs through IFN-γ production, which results in nitric oxide generation and tumor cell-killing effects (79). A T cell’s adaptive immune response is triggered by antigens presented by activated killer DCs (80). M1 macrophage polarization is notably induced by Th1 cell-derived IFN-γ, and this polarization is dependent on the interaction between Th1 cells and macrophages (81). Instead, Th2 cells create a positive feedback loop by inducing macrophages to become M2 polarized and promoting the proliferation of cancer cells and the secretion of IL-4 through the production of IL-10 and IL-4 (82). Furthermore, there is a positive association between Th2 cytokine levels and the quantity of MDSCs, and this indicates that patients with malignancies have a higher risk of dying (83). Th1 and Th2 cytokines and transcription factors work in feedback loops to maintain the balance between Th1 and Th2 cells (84). Th2 cytokine IL-4 is directly negatively impacted by IFN-γ, but effector Th1 cells are prevented from producing IFN-γ by IL-4 (85, 86). Furthermore, Th1 cells’ transcription factor T-bet limits Th2 cell differentiation by suppressing GATA3 expression in addition to inducing Th1 differentiation (87). The clinical results of cancer patients are strongly correlated with the Th1/Th2 balance. Overall survival in tumor patients is prolonged when there is a shift in the Th1/Th2 balance toward Th1 dominance (88). On the other hand, a change in the Th1/Th2 ratio that favors the Th2 phenotype creates an immunosuppressive milieu that promotes tumor recurrence (89). As a result, altering the Th1/Th2 ratio in favor of the Th1 phenotype could offer a promising treatment for reducing carcinogenesis and improving cancer remission.

Th17 cells are a specific inflammatory lineage of CD4^+^ helper T cells that release IL-17, IL-17A, IL-17F, IL-21, IL-22, and CCL20 at high concentrations (90–93). Many researchers have proposed reasons for why Th17 cells either stimulate or block the growth and development of tumors. For example, they may stimulate tumor angiogenesis, which gives tumors a rich blood supply and, therefore promotes tumor growth, as is the case in melanoma, bladder cancer, hepatocellular carcinoma, and colorectal cancer (91–96). Nevertheless, there is proof that Th17 cells can also function as natural killer (NK) cells and cytotoxic T lymphocytes (CTLs) in the fight against cancer, as seen in cases of lung and ovarian cancer (94).

#### 2.1.2 Natural killer cells

##### 2.1.2.1 NK cell origin, function, and tumor recognition

Natural killer (NK) cells, which are essential for tumor defense, are produced from pluripotent hematopoietic stem cells in the bone marrow and evolve from lymphoid progenitors and NK/T cell precursors (97). NK cells can directly and non-specifically kill tumor cells, making them the body’s first line of defense against cancer cells. The receptors that are expressed on the surface of NK cells are primarily responsible for their function. These receptors fall into two primary categories: those that regulate NK activity, known as inhibitory receptors (KIRs) and activating receptors (KARs, for example) (97).

Upon infiltrating the TME, NK cells can target cancer cells through the “missing-self” mechanism. Normally, inhibitory receptors on NK cells bind to class I HLA (MHC I) molecules and suppress NK cell activation. Moreover, NK cells complement CTL activity by eliminating tumor cells that escape antigen-specific detection, thereby providing an essential second line of defense. However, many cancer cells downregulate MHC I expression to evade detection by CD8^+^ T cells, which makes them vulnerable to NK cell attack. Consequently, the absence of MHC I–mediated inhibitory signals shifts the balance toward activating signals, ultimately leading to the lysis of target cells (98). In addition, NK cells secrete pro-inflammatory cytokines such as IFN-γ and TNF in response to transformed cells; these cytokines not only exert potent anti-proliferative, anti-angiogenic, and pro-apoptotic effects on cancer cells but also enhance cytotoxic CD8^+^ T cell responses to stimulate adaptive immunity (99).

##### 2.1.2.2 NK cell subtypes and distribution in tumors

It is important to note that the role of NK cells is highly context-dependent. In humans, NK cells can be subdivided into two major groups based on the expression levels of CD56 (NCAM1) and CD16 (FCGR3A): the CD56^dim^CD16^hi^ population, which mainly mediates cytotoxicity by secreting perforin and granzymes, and the CD56^bright^CD16^lo^ population, which predominantly exhibits immunoregulatory and cytokine-producing functions. Recent research shows that the immature CD56^bright^CD16^lo^ NK cells were largely predominant in nasopharyngeal cancer and basal cell carcinoma, whereas the mature CD56^dim^CD16^hi^ NK cells occupied renal carcinoma and lung cancer, consistent with previous reports. In other tumor types, such as colorectal cancer and HCC, no obvious propensity is observed. Moreover, a pan-cancer study identified a tumor-enriched NK cell subset—tumor-associated NK (TaNK) cells—which display elevated stress-response genes (e.g., DNAJB1/HSP40) and reduced levels of cytotoxic molecules (e.g., granzyme B, perforin). Notably, TaNK cells express higher levels of inhibitory receptors (e.g., KIRs) and lower levels of cytotoxic markers, suggesting a potentially “dysfunctional” phenotype with impaired tumor-killing capacity (100).

##### 2.1.2.3 Tumor microenvironment effects on nk cell function

The TME itself poses significant challenges to NK cell function. Due to vascular abnormalities and increased local oxygen demand, the TME of solid tumors is often hypoxic (101, 102). This is hazardous to lymphocytes that infiltrate the tumor, especially NK cells (103, 104). Hypoxia induces a transcriptional rewiring in NK cells, leading to the downregulation of various effector molecules and activating receptors, while inhibitory receptors, cytokine receptors, or receptors mediating ADCC are less affected (Box 1) (105–108). In addition, several mechanisms operating within the TME alter NK cell function. For example, patients with prostate cancer exhibit significantly lower NK cell IFN-γ production and a reduced proportion of CD56bright cells compared to controls (109). Similarly, in hepatocellular carcinoma (HCC) tissues, NK cells show impaired IFN-γ production and diminished cytotoxicity (110). In studies involving pancreatic and breast cancers, NK cells have been found to express high levels of inhibitory receptors (such as NKG2A) and low levels of activating receptors (including NKG2D, NKp30, NKp46, CD16, and DNAM1), which correlates with reduced cytotoxic function (111). In this context, activation of NK cells is initiated by the phosphorylation of immunoreceptor tyrosine-based activation motifs (ITAMs) in adapter proteins (112), while a diverse repertoire of inhibitory receptors provides self-tolerance and negative-feedback mechanisms (113).

In addition to hypoxia, the TME’s acidic and anoxic conditions—largely a consequence of the Warburg effect (49)—further impair NK cell function. NK cells are extremely sensitive to increased lactic acid, which inhibits the upregulation of nuclear factor of activated T cells, thereby compromising their function and survival. High extracellular lactic acid is taken up by NK cells, resulting in decreased intracellular pH, reduced ATP concentration, and impaired energy metabolism; these changes collectively lead to diminished NK cell activity and enhanced apoptosis (114). Concurrently, adenosine levels rise during tumor hypoxia, impairing NK cell metabolism by inhibiting both oxidative phosphorylation (OXPHOS) and glycolysis, and thereby undermining the metabolic pathways activated by IL-12 and IL-15 (115). Moreover, adenosine has been demonstrated to suppress both cytokine production (116) and cytotoxic activity (117, 118) in activated NK cells through an A2AR-initiated, cAMP-dependent pathway involving protein kinase A (PKA) (116–118). Notably, CD56brightCD16lo NK cells express CD38 and ENPP1—key components for adenosine synthesis—resulting in abundant adenosine production that can exert potent immunosuppressive effects on other immune effectors such as CD4^+^ helper T cells (119). Although IL-18 signaling typically induces KIT expression to promote tumor growth (120), A2AR-deficient mature NK cells show reduced IL-18R1 and KIT expression, making them less susceptible to adenosine’s inhibitory effects (121). Recent tumor models treated with A2AR antagonists have shown significant inhibition of spheroid growth in primary breast cancer patients (122), suggesting that adenosine and its related pathways may be promising targets for future immunotherapy.

Beyond metabolic challenges, hypoxia-inducible factor 1α (HIF-1α) also plays a crucial role in modulating immune responses within the TME. While preclinical studies initially demonstrated that HIF-1α inhibits CD8^+^ T cells and promotes the conversion of tumor-associated macrophages (TAMs) to the M2 phenotype, its effects on NK cells remained unclear. However, single-cell RNA sequencing in a human lymphoma cell model revealed that conditional deletion of HIF-1α in NK cells leads to slower tumor progression, increased expression of activation markers and effectors, and enrichment of the NF-κB pathway in tumor-infiltrating NK cells. Importantly, IL-18 is necessary to activate NF-κB and boost the antitumor activity associated with HIF-1α; in its absence, NK cells lacking HIF-1α cannot effectively control tumor progression (123). Similarly, in a B16-F10 melanoma model, blocking HIF-1α transcription significantly inhibited tumor development and promoted the infiltration of both CD8^+^ T cells and NK cells into the TME via increased release of chemokines CCL2 and CCL5 (61). These findings underscore the pivotal role of HIF-1α in regulating immune cell transformation and death, with potential implications for future immunotherapeutic strategies.

##### 2.1.2.4 Therapeutic approaches and future perspectives

Moreover, recent studies have found that the surface membrane protrusions of NK cells in the TME are markedly reduced compared to those in normal tissues, thereby diminishing their ability to interact with and kill tumor cells. This reduction is likely due to disrupted serine metabolism in tumors, which leads to decreased sphingomyelin (SM) levels in NK cell membranes. Notably, treatment with antagonistic sphingomyelinase significantly reduces tumor cell numbers, and combining this approach with other immunotherapies may facilitate rapid clinical implementation (124).

In parallel with these natural mechanisms, engineered NK cells—such as CAR-NK cells—are emerging as a promising immunotherapeutic tool. CAR-NK cells have been widely applied in hematological malignancies like multiple myeloma and B cell lymphoma. For instance, a viral construct of CS1-specific CAR, which targets a protein highly expressed in multiple myeloma cells, was successfully used in both *in vivo* and *in vitro* studies, resulting in enhanced IFN-γ expression and activated cytolysis of myeloma cells (125). Similar beneficial outcomes have been observed with CD22-specific CAR-NK cells in treating B cell lymphoma (126). In addition, CAR-NK cell therapy is being explored in solid tumors such as breast cancer, glioblastoma, and melanoma (127). Besides the “missing-self” mechanism and cytokine production, therapeutic monoclonal antibodies targeting tumor-associated antigens can harness NK cell-mediated antibody-dependent cellular cytotoxicity (ADCC), which plays a critical role in the effectiveness of agents like trastuzumab and rituximab against both solid and hematological malignancies (128).

Finally, it is noteworthy that while many T cell–focused immunotherapies target PD-1 or CTLA-4, NK cells in many solid tumors do not express these molecules at high levels. Instead, NK cell suppression is often mediated by other checkpoint molecules (e.g., TIGIT) and various signals within the TME. This has sparked debates regarding how best to harness checkpoint pathways for NK cell–based therapy. At the same time, cytokines such as IL-15—often produced by specialized dendritic cells like LAMP3^+^ DCs—are crucial for maintaining NK cell cytotoxicity; however, these signals are frequently dysregulated in tumors. Therefore, it is essential to consider tumor type–specific or subset-specific contexts to effectively “revitalize” NK cells. In this regard, distinguishing tissue-infiltrating NK cells from those in peripheral blood is also critical. For example, markers such as RGS1 have been found to reliably differentiate NK cells that have entered tumor or healthy tissues from those circulating in the blood. Clinically, a higher proportion of these tumor-associated (or dysfunctional) NK cells is often linked to poorer patient outcomes and resistance to certain immunotherapies, suggesting that strategies aimed at reversing NK cell dysfunction (e.g., via IL-15 administration or TIGIT blockade) may be promising. In summary, a nuanced discussion of NK cell biology—whether by examining specific subsets (e.g., CD56^bright^CD16^lo^ versus CD56^dim^CD16^hi^) or by focusing on particular tumor contexts—can provide greater clarity and help address ongoing controversies in the field (99).

#### 2.1.3 M1 macrophages

Macrophages are an essential component of TME; making up nearly 50% of the mass of TME in some solid tumors (129). TME and other stimuli can differentiate between macrophages1 (M1) and M2 types of macrophages, with M1 being connected to antitumor immunity and M2 to immunosuppression. In early tumor stages, IFN-γ, TNF-α, and lipopolysaccharide induce polarization of the M1 phenotype. M1 can secrete reactive oxygen species, interleukins, TNF-α, and NO, which increase macrophage phagocytosis activity and promote T-cell activation. As the tumor progresses, in the TME, nutrients decrease, adenosine and lactate increase, and hypoxia and pH decrease, which induce macrophages to exhibit an M2 phenotype. M2 macrophages can express IL10, IL-12, arginase 1, etc., and these promote Treg cell recruitment, epithelial-mesenchymal transition (EMT), extracellular matrix (ECM) remodeling, angiogenesis, and finally, tumor metastasis (7, 97, 130).

C7-APOBEC3A M1 macrophages were primarily found in early stage 1 of high-grade serous ovarian cancer (HGSOC) and showed increased chemokine secretion activities, according to a scRNA-seq study. C7-APOBEC3A M1 macrophages were consistently linked to improved survival rates (131). In another study, Tumor-infiltrating macrophages (TIMs) may change from M2 to M1 in response to NF-κB subunit p50 reduction if NF-κB activation is blocked (132). Moreover, TIM derived from p50-deficient animals recovered canonical NF-κB activity together with an M1 phenotype linked to tumor reduction (132). A thorough investigation showed that bufalin targeted p50, whose overexpression was inhibited by bufalin-induced ubiquitination, resulting in compensatory creation and p65-p50 translocation into macrophage nuclei. The production of immunostimulatory cytokines was facilitated by the rise of p65-p50 in the nuclei, which in turn triggered the immunological response of T cells against tumors (133). Carfilzomib stimulated NF-κB to transcribe the M1 marker-encoding gene in M2 macrophages in another experiment and mouse lung cancer model. In a mouse model, a synergistic impact of Carfilzomib and anti-PD-1 resulted in an almost complete regression of primary lung cancer (134). As mentioned above, the combination of anti-PD-1 and anti-CTLA-4 therapy has strong side effects. Conducting future experiments to assess the side effects of Carfilzomib and anti-PD-1 can benefit patients.

Macrophages are typical in malignancies. Hence, CAR-macrophage treatment offers excellent potential. There have been reports of the production and characterization of anti-HER2 CAR macrophages based on CD3ζ (135). A replication-deficient adenovirus vector efficiently and repeatedly delivers CAR to macrophages. Infection with adenovirus produces M1 differentiation of CAR macrophages and the pro-inflammatory state of the TME. Furthermore, as professional antigen-presenting cells, adenovirus-transduced CAR macrophages can present tumor-derived antigens and target antigens to activate more T cells.

Moreover, these CAR macrophages significantly prolonged the survival of mice with tumor implants and decreased lung metastasis. Nevertheless, *in vitro* cultures of CAR macrophages are expensive. In contrast, the macrophage targeting nanocarrier and the nanocomposites of CAR-INF- encoded plasmid DNA may significantly lower this cost, and CAR-M1 macrophages can be activated subsequently *in vivo* to phagocytize CAR-mediated malignancy, regulate antitumor immunity, and prevent the formation of solid tumors (136). While it has not yet been applied clinically, this approach could address limitations in CAR-NK and CAR-T therapy, potentially significantly extending the lifespan of more tumor patients.

#### 2.1.4 Neutrophils1

In 2009, researchers reported for the first time that neutrophils in TME might also be divided into antitumor neutrophils1(N1) and tumor-promoting neutrophils2(N2) (137). CD206 can be used as a mark to distinguish between N1 and N2 in the clinical setting (138). Traditionally, neutrophils are fully differentiated cells with a short lifespan, while tumor-associated neutrophil (TAN) has a longer lifetime (139). N1 can play an antitumor role indirectly through T, B, NK, and other cells (140, 141), and directly inhibit tumors by enhancing the activity of NADPH oxidase, which produces reactive oxygen species (ROS) (142). Besides, *in vitro* experiments showed that N1 had high ICAM-1 and FasR levels and secreted a large amount of IP-10 and TNF to recruit NK cells and promote DC antigen presentation to T cells (140, 141). During the growth of the tumor, the early neutrophils are mainly located at the margin of the tumor and have a predominant N1 phenotype. N1 and N2 can be switched to each other. Previous research demonstrated that TGF-β is closely related to N2 polarization (143, 144), while INF-β or blocking TGF-β can stimulate tumor-associated neutrophils (TAN) to polarize to N1 (145). As the tumor progresses, neutrophils migrate to the tumor center and express the N2 phenotype. In addition, the analysis of postoperative specimens from patients with advanced gastric cancer demonstrated that N2 and Tumor-Stroma ratio in the tumor center was substantially associated with poor disease-free survival (146). A regression analysis also suggested that the OS and relapse-free survival (RFS) of the high N1 infiltration group in TME were considerably longer than those of the low infiltration group. In addition, multivariate analysis revealed that a high N1/N2 ratio was an important prognostic index for OS and RFS (142). The effect of exosomes that inhibit the migration of N1 to the tumor center or block the secretion of tumor cells on TAN may be the focus of research in the future.

#### 2.1.5 B cells

The B cells in TME also have a unique character in antitumor immunity. According to earlier studies, about 35% of lung cancers showed signs of growing B cells. Furthermore, TIL-B (tumor-infiltrating B lymphocytes) are present at every stage of the development of human cancer (147). Tumor-infiltrating B cells are implicated in anticancer responses as potent, diverse players, according to an abundance of recent evidence. Exhausted or dysfunctional CD8^+^ and CD4^+^ TILs exhibit the B cell-recruiting C–X–C pattern chemokine ligand 13 (CXCL13), which often indicates that B cells are designed to provide assistance in the face of tumor persistence (148–150). By means of scRNA-seq, plasmablast-like TIL-Bs from patients suffering from melanoma and ovarian cancer demonstrated increased expression of transcripts encoding IFNγ and chemokines that draw T cells, macrophages, and natural killer cells (like CCL5, CCL4, and CCL3) in comparison to other TIL-B subsets, and were in fact linked to increased T cell infiltration (151, 152). Apart from the cytokine production function, A recent investigation on mice employing model antigens demonstrated evidence of antigen presentation by B cells specific to malignancies, which resulted in increased CD8^+^ T cell and TFH cell responses against tumors (153). Furthermore, the release of IL-10 has been shown as the most often occurring Breg cell effector mechanism in human cancer (154). IL-10^+^ Breg cells have been identified using immunohistochemistry or flow cytometry in a number of human malignancies, including as tongue squamous cell carcinoma (155), gastric cancer (156), and breast cancer (157). Furthermore, through a mechanism similar to antibody-dependent intracellular neutralization—a process by which antibodies induce the proteasomal degradation of intracellular proteins (158, 159)—it was demonstrated that TIL-B-derived IgG from lung cancer promoted the degradation of the tumor protein Ras homolog family member C (160). Furthermore, B lymphocytes play a crucial role in immunotherapy, and their presence has been related to a better prognosis in various cancer types. PD-1 and PD-L1 targeting immune checkpoint inhibition has been shown to directly influence TIL-Bs, as evidenced by the presence of PD-1^+^ and PD-L1^+^ TIL-Bs in a number of human malignancies, including breast cancer and hepatoma (161–164). Patients with melanoma, sarcoma, and lung adenocarcinoma are among those whose baseline TIL-B density predict their response to PD-1 and CTLA4 inhibition (165–168), and are in fact strengthened by immune checkpoint inhibition (166, 167, 169). In a mouse model of breast cancer, B cells were necessary for responding to CTLA4 and PD-1 inhibition. In a squamous cell carcinoma model, the addition of an anti-PD-L1 antibody to radiation therapy changed a Bregcell response to an effector B cell response linked to better tumor control (170). In addition, B cells can reside in tertiary lymphoid structures (TLS) within the tumor and promote T cell activation through antigen presentation. However, B cells, especially regulatory B cells, can produce IL-10, TGF-β, and pro-angiogenic mediators, promoting an immunosuppressive phenotype in neutrophils, macrophages, and CTLs and supporting tumor growth (7, 9).

Further studies also showed a significant decrease in antibody production after blocking the membrane B cell activator (BAFF) receptor. Therefore, the membrane BAFF on TAN is a potential contact mechanism (171) for mediating B cell differentiation and may serve as a future immunotherapy target. Besides, blocking the BAFF receptor can also increase antibody release, which can be directed against specific tumors by specific antibodies. In a mouse model of colon cancer, they improved survival in mice with colorectal cancer with an Asian-specific variant of human IgG1 containing the Gly396 to Arg396 substitution (hIgG1-G396R). Immunoassays with TME demonstrated an antagonistic effect on the active tertiary lymphoid structure and promoted tumor-associated antigen-specific (TAA-specific) plasma cell differentiation to produce antibodies. Adoptive metastasis of TAA-specific class-switched memory B cells carrying this variant shows therapeutic effects in a mouse tumor model (172). In addition, researchers have found that in tumor tertiary lymphoid structures, plasma cells producing IgG and IgA are spread into tumor beds along fibroblast pathways. Treatment response and progression-free survival are associated with IgG-stained tumor cells in renal cell carcinoma patients treated with immune checkpoint inhibitors (173). They can antagonize tumors by increasing the number of antibodies or specific antibodies against different tumors. Although there are few clinical applications for now, they have a good prospect.

#### 2.1.6 Conventional dendritic cells

The leaders of the human immune system, dendritic cells (DCs) can present, process, and phagocytose antigens to improve immunity against tumors. DCs come in three varieties: monocyte-derived DCs (moDCs), plasma DCs (pDCs), and conventional DCs (cDCs). Among them, conventional type I DCs (cDC1) and conventional type II DCs (cDC2) are two subtypes of cDCs that contribute to the advancement of anti-tumor therapy. By cross-presenting tumor-associated antigens to CD8^+^ T cells, cDC1s, which are experts in intracellular antigen processing and presentation, produce anti-tumor immune responses. cDC2s effectively elicit Th1, Th2, and Th17 polarization in CD4^+^ T cells by presenting MHC II-associated antigens to them (174, 175).

DCs are the immune system’s most potent antigen-presenting cell (APC) and one of the main players in the adaptive immune response out of all the immune cells. The upkeep and initiation of anticancer immunity depend heavily on DC-mediated cross-initiation of tumor-specific T lymphocytes. Their presence in tumors will increase patient survival, stop the spread of malignancy, and activate T-cell responses. The expression of MHC-II molecules and integrin-αX (CD11c) characterize the phenotype of cDC. They may reside in lymph nodes or migrate from peripheral tissues carrying antigens. There is increasing evidence today that the cross-initiation of tumor-resident cDC is necessary to generate an antitumor immune response (176). Most existing drugs that enhance the antitumor activity of cDC can also stimulate other antitumor immune cells. Previous studies have found that tumor-derived prostaglandin E2(PGE2) inhibits the IL-12 production of cDC1, downregulates the expression of costimulatory molecules, and decreases the induction of antitumor responses. A previous experiment has mentioned that MF-766 is an effective and highly selective small molecule that inhibits EP4 receptors. Through multi-parameter flow cytometry analysis, some researchers have found that treatment with MF-766 promotes the infiltration of CD8^+^T cells, NK cells, and cDC, which induces the reprogramming of M1^-^like macrophages and decreases the myeloid-derived suppressor cells (MDSC) in TME (177). Similarly, in another experiment, tumor growth was significantly inhibited by the injection of CpG oligonucleotide-B (CpG-B) into an established solid tumor model. Tumor growth inhibition following injection of CpG-B depends on neutrophil recruitment to the environment, which activates cDC, followed by increased antitumor T^-^cell initiation in the draining lymph nodes and increased effector T-cell infiltration into the tumor microenvironment (178). In addition, Single-cell RNA sequencing (scRNA-seq) is frequently employed to assess the diversity of tumor cells. In recent years, researchers selected colon cancer patients’ immune and stromal populations using scRNA-seq. They found that treatment with a CD40 agonist antibody could activate the cDC and increase the number of Bhlhe40^+^Th1^-^like cells and CD8^+^ memory T cells, thereby enhancing the antitumor effect (179). Thus, cDCs are involved in the anti-tumor or pro-tumor processes of many drugs. However, there is also experiment showed the inability of tumor-bearing mice’s dendritic cells to induce an immune response has been noted by scholars (180). Thus, the mechanism of the role of cDCs in anti-tumor needs to be further explored.

Except cDCs, moDCs also exhibit the ability of antitumor. Those cells are induced to transfer into TNF/iNOS-producing DCs (Tip-DCs) in the inflammatory environment and have been proven to produce NO for CD8^+^ T cells to kill tumor cells in the murine model (150). However, another study indicates that the NO produced by Tip-DCs contributes to immunosuppressive effects (181). Considering its controversial properties, more explorations are needed to explain the role of moDCs in TME.

### 2.2 Tumor-promoting immune cells

#### 2.2.1 Regulatory T cells

Regulatory T cells (Treg) have immunosuppression and tumor growth promotion functions (182, 183). Treg relies on cyclic-coupled oxidative phosphorylation of tricarboxylic acids and fatty acid oxidation to support its survival and differentiation. Treg can be divided into natural Treg (nTreg) and *in vitro* induced Treg (iTreg), with iTreg suppressing the antitumor immune effect in the tumor microenvironment (184).

Recent studies have revealed that the role of Treg cells in tumors is highly heterogeneous. In many cancers—such as melanoma, non‐small cell lung cancer, and head and neck cancers—effector Tregs (commonly classified as Fr. II cells) accumulate and express elevated levels of immunosuppressive molecules including CTLA-4, PD-1, CCR4, and CCR8. This phenotype not only intensifies local immunosuppression but also supports tumor immune evasion. In contrast, in tumors like colorectal and certain head and neck cancers, a higher proportion of non-suppressive Foxp3^+^ cells (often termed Fr. III cells) correlates with improved patient outcomes, underscoring that both the quantity and functional state of Treg infiltration are critical determinants of prognosis (185).

Moreover, the downstream signaling pathways that mediate Treg suppressive function are multifaceted. Treg cells execute suppression via CTLA-4–dependent transendocytosis of the co-stimulatory molecules CD80/86, high-affinity IL-2 consumption through CD25, and secretion of inhibitory cytokines such as IL-10, TGF-β, and IL-35. In addition, they modulate the tumor microenvironment by reprogramming local metabolism—utilizing lactate and generating immunosuppressive adenosine through the concerted actions of CD39 and CD73. These mechanisms complement the intrinsic metabolic programming based on oxidative phosphorylation and fatty acid oxidation that underlies Treg survival and function (185, 186).

Immunotherapeutic strategies targeting Treg cells have also advanced considerably. While conventional immune checkpoint blockade (ICB) with anti-CTLA-4 and anti-PD-1 antibodies aims to reinvigorate effector T cells, these agents can also modulate Treg activity within tumors. Novel approaches—such as bispecific antibodies designed to simultaneously target CTLA-4 and PD-1, as well as therapies directed against CCR8, GITR, and TIGIT—seek to selectively deplete or reprogram tumor-infiltrating Tregs. Such strategies promise to restore effective antitumor responses while minimizing systemic autoimmunity (185).

Besides, studies have found that iTreg cells can promote tumor immune escape by inhibiting cytotoxic T lymphocytes redirected by immune-mobilizing monoclonal T-cell receptors against cancer-NYE (187). In the tumor microenvironment (TME), 5%–30% of CD4^+^Foxp3^+^ Treg cells have been observed to highly express granzyme B—a feature absent in Treg cells from some mouse models—suggesting that granzyme B production enables Tregs to kill NK cells and CD8^+^ T cells. Conversely, the utility of RGB has been shown to selectively suppress the differentiation of CD4^+^Foxp3^+^ Treg cells, thereby inhibiting tumor growth (188, 189).

Recently, to elucidate the contribution of Treg-mediated immunosuppression in the TME, researchers depleted Treg cells in a pancreatic cancer mouse model. Unexpectedly, the absence of Tregs failed to relieve immunosuppression and instead accelerated tumor progression (190). There is some evidence that Treg depletion may have triggered a pathological T-cell response—characterized by a Th2-type cytokine profile—since both Th2 and Th17 cells are implicated in pancreatic cancer development (191, 192). Therefore, given that pancreatic ductal adenocarcinoma (PDA) responds poorly to T-cell immunotherapy, it is possible that other processes (such as impaired induction of T cells by dendritic cells) are concurrently inhibited (193).

Furthermore, recent findings indicate that after Foxp3^+^ Treg depletion, CD4^+^Foxp3^–^ T cells may acquire the capacity to suppress antitumor immunity; however, blockade of IL-10 signaling in conjunction with Treg depletion can overcome this treatment resistance (194). Another study demonstrated that in a glioma mouse model receiving radiotherapy and chemotherapy, administration of NT-I7 (a long-acting IL-7) resulted in an increase in T lymphocytes across lymph nodes, thymus, and spleen, enhanced production of IFN-γ, and a reduction in intratumoral Tregs, which correlated positively with survival rates (195). Although reports remain mixed, these observations suggest that maintaining Treg infiltration within a specific range in the TME may be critical for optimizing patient benefits.

#### 2.2.2 M2 macrophages

In tumor proliferation and metastasis, IL-13, IL-4, and IL-10 in TME induce macrophages to polarize to the M2 phenotype (196). The polarized M2 macrophages can increase phosphoglycerate kinase 1 (PGK1) phosphorylation in tumor cells, mediated by 3-adenosine phosphate-dependent protein kinase 1 (PDPK1) in tumor cells by secreting IL-6. This phosphorylation enhances PGK1-catalyzed glycolysis by altering substrate affinity, thereby increasing tumor energy supply (197). The long-term hypoxia, nutrient deficiency, and a high concentration of lactic acid in TME are the critical factors for inducing tumor-associated macrophages to display an M2-like phenotype (198, 199). KRAS gene can stimulate the production of CSF2 and lactic acid in tumor cells by stabilizing HIF-1α in human colorectal cancer specimens (200). However, high lactic acid in TME induces enhanced expression of Arg1 and vascular endothelial growth factor in macrophages through the HIF-1α-mediated signaling pathway, resulting in an M2-like phenotype (201).

Autophagosomes (TRAPs) released by tumor cells are a new mechanism for immunosuppression. They are readily absorbed by B cells and then induce the production of IL-10, which may inhibit T cell proliferation and antitumor response (202) and can also induce M2 polarization via the increased IL-10 and PD-L1 expression. These macrophages inhibit CD8^+^ and CD4^+^T cell proliferation *in vitro* and enhance tumor growth by PD-L1 *in vivo* (203). In addition, prior research indicates that PD-L1 inhibitors can promote the development of tumor-associated macrophages to the M1 state in glioma (204, 205) and analysis of glioma data from the TCGA and CGGA databases found that a higher expression of PD-L1 resulted in a shorter overall survival. By inhibiting the expression of PD-L1 and subsequently blocking the polarization of macrophages, the infiltration of associated immune cells might be significantly increased to enhance their tumor-killing effect.

#### 2.2.3 Tumor-associated macrophages

Macrophages infiltrating TME are tumor-associated macrophages (TAM) (206). In solid tumors, TAMs are highly enriched within the TME, where they are “co-opted” by cancer cells to support tumor progression. For example, tumor cells release autophagosomes (TRAPs) and an LC3-II+ double-membrane extracellular vesicle (EVs). TRAPs further reduce the antitumor response by polarizing TAMs to the M2 phenotype via the TRAPs–PD-L1 axis (194). In addition, cancer cells secrete succinic acid in the TME, which activates the succinic acid receptor (SUCNR1). The SUCNR1-triggered PI3K–HIF-1α signaling cascade promotes tumor cell migration and invasion (207). Moreover, in gliomas and other solid neoplasms, tumor-derived kynurenine activates aryl hydrocarbon receptors (AHR) in TAMs. This not only upregulates CCR2 expression—enhancing TAM recruitment via the CCL2 axis—but also induces the expression of CD39, which cooperates with CD73 to produce adenosine that impairs CD8^+^ T-cell function (208, 209). Tumors enhance immune escape by metabolically reprogramming immune cells, which may offer new therapeutic opportunities in the future.

Furthermore, metabolic reprogramming plays a critical role in shaping TAM functions in solid tumors (210). Owing to the competition and consumption of energy by tumors, immune cells in TME frequently acquire energy from substances other than sugars. For instance, through the observation of pathological tissue sections, scholars have revealed that the lipid content of TAMs in multiple myeloma (MM), breast cancer, colon cancer, and prostate cancer is significantly higher than that of macrophages in control groups. Further cell culture and *in vitro* experiments in tumor-bearing mice showed that blocking or knocking out CD36 inhibits lipid uptake by TAMs and stops their pro-tumoral functions (211). CD36 also plays an important role in myeloid-derived suppressor cells (MDSCs) by enhancing fatty acid absorption and oxidation, thereby acquiring energy to support their immunosuppressive function (212). Additionally, elevated levels of lactic acid in the extracellular environment, caused by anaerobic glycolysis of tumors, drive metabolic shifts in TAMs. MHCIIlo TAMs can transition from aerobic glycolysis to utilizing lactic acid as a carbon source, fueling the tricarboxylic acid (TCA) cycle (213), increasing L-arginine metabolism, and further enhancing TAM-mediated suppression of T cells (214, 215).

In contrast, while TAMs in hematologic malignancies such as Hodgkin lymphoma also contribute to an immunosuppressive microenvironment, their roles tend to be shaped by the unique milieu of bone marrow and lymphatic tissues. For instance, high infiltration of CSF1R-positive, CD68-positive macrophages in Hodgkin lymphoma is closely linked with poor progression-free survival, suggesting that in these blood cancers TAMs are primarily involved in sustaining malignant cell survival and immune evasion rather than promoting processes like angiogenesis or invasion that are more characteristic of solid tumors. Additionally, the metabolic reprogramming observed in hematologic contexts may differ in degree and substrate utilization, reflecting the distinct nutrient and cytokine landscapes in these tissues (216).

Earlier research has revealed that substantial TAM infiltration is strongly associated with a poor prognosis in most tumors (217). This may be associated with TAM subgroups. In addition to M2, some macrophages express the scavenger receptor MARCO, which is related to a poor prognosis in glioma patients (218). The mechanism may involve increased tumor vascularization and the reduction of the tumor-killing effect of NK cells by inhibiting TNF-related apoptosis-inducing ligand (TRAIL) (219). Moreover, MARCO-expressing macrophages inhibit NK and T cells’ cytolytic activity and antitumor capability by secreting IL-37 and upregulating Treg cell activity (220). The interaction between tumors and TAMs is observable, and disrupting either may lead to improved therapeutic outcomes.

Alongside these insights, emerging immunotherapeutic strategies are increasingly focusing on TAM-targeting as a means to overcome tumor-induced immune suppression. For example, checkpoint inhibitors—such as anti-PD1/PDL1 and anti-CTLA4 antibodies—have been shown to partially restore cytotoxic T-cell activity; however, their efficacy might be enhanced when combined with agents that specifically modulate TAM functions. Notably, therapeutic approaches include CSF1R inhibitors (e.g., PLX3397 or pexidartinib) that deplete pro-tumoral TAMs or reprogram them towards an antitumoral M1 phenotype and novel strategies employing engineered macrophages (CAR-M) or nanoparticle-mediated drug delivery to further exploit TAM plasticity. These combination therapies are designed to dismantle the immunosuppressive networks within the TME, thereby potentiating conventional therapies such as chemotherapy and radiotherapy (210, 216).

In summary, while TAMs in solid tumors predominantly facilitate tumor progression through a network of metabolic, migratory, and immune-suppressive pathways (including the TRAPs–PD-L1, SUCNR1–PI3K–HIF-1α, and AHR pathways), those in hematologic malignancies tend to reinforce malignant cell survival and immune evasion within distinct microenvironments. Concurrently, immunotherapeutic strategies targeting these cells—by either depleting, re-educating, or blocking their recruitment—offer promising avenues to overcome treatment resistance and improve overall patient outcomes.

#### 2.2.4 Neutrophils2 and tumor-associated neutrophils

As introduced above, N2 has the effect of tumor-promoting in the TME. N2 neutrophils in CTC clusters support tumor progression, promoting the formation of metastatic niches and the proliferation of circulating tumor cells (CTCs), both circulating and upon arriving at distant organs. N2 neutrophil depletion or augmentation results in delayed or accelerated metastasis development, respectively (221). N2 neutrophils promote Treg recruitment, inhibit NK cells, alter the extracellular matrix, release VEGF, and stimulate angiogenesis, thereby promoting tumor growth (7, 143). N2 neutrophils in the pre-metastatic niche have been shown to be able to suppress T cell activity in another investigation. It was discovered that PD-L2 was in charge of the T-cell suppression brought on by N2 neutrophils. In order to induce PD-L1 and gain the ability to suppress T-cell activity, N2 neutrophils were stimulated by GM-CSF (222) or IL-6 (223, 224). However, investigations have demonstrated that early TAN in TME did not exhibit immunosuppression. In contrast, it stimulated T cells to inhibit tumor growth, consistent with the theory above of TAN migration in tumors. It was found that 5%-25% of cells extracted were TANs in the digested human lung tumors, which presented activated phenotype with a distinct repertoire of chemokine receptors and produced enormous proinflammatory factors as well as the antiinflammatory factors antagonist that normal neutrophils (225). In addition, like normal neutrophils, TANs could also stimulate the proliferation of T cells and the excretion of IFN-γto inhibit the progression of tumors (122). Similarly, Gastric cancer cell-derived exosome (GC-Ex) can activate neutrophil autophagy and promote tumor through the HMGB1/TLR4/NF-κB signaling pathway, which may be one of the underlying mechanisms for promoting TAN migration by TME (226). Another study has found that in breast cancer, extracellular vesicles (EVs) induce N2-like phenotype neutrophils, increase migration, release NETs, ROS, IL-8, and VEGF, and elevate arginine-1 expression, thereby improving the viability of breast tumor cells (227). A similar phenomenon was observed in melanoma, where EVs increased the expression of N2 molecular markers (such as VEGF, ARG1, and CXCR4) in neutrophils, prolonged TAN life, and induced neutrophils into a neoplastic state (228). Although N1 and N2 have only been proposed recently, they have taken a certain direction in cancer treatment.

#### 2.2.5 Myeloid-derived suppressor cells

Myeloid-derived suppressor cells (MDSC), arguably the most important tumor-sparing cells of TME, were first described in 2007 (229). MDSCs are produced under chronic inflammatory conditions and are characterized by the continuous production of inflammatory cytokines and chemokines, such as IL-6. IL-6 upregulates CCR5 in MDSCs via the STAT3 pathway, whereas CCR5 is crucial in promoting tumor recruitment and activation of MDSCs (230). The differentiated MDSCs in the presence of IL-6 strongly inhibit CD8+T-cell function (231). Additionally, they produce arginase and nitric oxide, expand Treg populations, and inhibit T cells, NK cells, DCs, and other cells via paracrine and cell-cell contact, suppressing antitumor immune responses (8, 9). Interferon gene stimulators (STING) have always been a topic of intense interest in cancer research. It is regarded as one of the most critical regulatory factors for antiviral and antitumor immunity. In a nasopharyngeal carcinoma (NPC) model, STING increased SOCS1 expression in tumor cells and MDSCs, inhibiting NPC-derived MDSC induction. SOCS1 prevents phosphorylation and dimerization of STAT3, which is a crucial inducer in the process of MDSCs expansion and is modulated by many factors like the granulocyte-macrophage colony-stimulating factor (GM-CSF), granulocyte colony-stimulating factor (G-CSF) (232). by physical interaction with the STAT3, thereby reducing the installation of MDSC by inhibiting the production of GM-CSF and IL-6 (233). In addition, MDSCs are not formed entirely within the tumor and need to pass through the circulatory system to be recruited in the tumor. Intervention with MDSC precursors during transport can indeed achieve the purpose of reducing MDSC recruitment and slowing tumor progression. PKR-like endoplasmic reticulum kinase (PERK) mediates the reprogramming of HSPC into a stereotypical MDSC precursor in the spleen by the PERK-ATF4-C/EBPβ signal. In addition, the absence of PERK converts MDSC to myeloid cells, activating CD8^+^ T-cell-mediated antitumor immunity.

Furthermore, a decrease in NRF2 signaling in PERK-deficient MDSC caused an increase in cytoplasmic mitochondrial DNA, leading to a dependent expression of antitumor Type I interferon (234). Interestingly, in recent years, several scientists have proposed IDO vaccination therapy against MDSC. This vaccine can achieve specific effectiveness in TME regardless of whether it is IDO^+^ or IDO^-^. IDO vaccine treatment activated CD8þ T-cells enriched with CD80 dendritic cells and killed the IDO^+^ cells (235).

#### 2.2.6 Plasmacytoid dendritic cells

Plasmacytoid dendritic cells (pDCs) stimulate and maintain pro-tumor immunity. In hepatocellular carcinoma, HIF-1α transcription upregulates CD39 and CD73 expression. In addition, it activates downstream extracellular adenosine (eADO) binding to adenosine A1 receptor (ADORA1) to enhance pDCs recruitment into the tumor. Besides, eADO-stimulated pDCs promote the induction of regulatory T cells and inhibit CD8^+^T cell proliferation and cytotoxicity (236). The infiltration of pDCs is also proved to be negatively related to the prognosis of ovarian cancer, melanoma, hepatocellular carcinoma, and many other tumors (236, 237). Moreover, tumors can secrete substances that impair pDC’s ability to produce type I interferon via the TGFβ-β pathway and subsequently inhibit its ability to activate the NK cells, proliferation of Th1 cells and inhibit the function of Treg (238, 239). In addition, in the colon cancer model, innate lymphocytes (ILCs) were negatively correlated with the pathological staging of the tumor. pDCs can induce apoptosis of ILCs through the CD95 pathway in a tumor-like microenvironment, leading to tumor progression (240). Interestingly, pDCs have an anti-tumor effect as well. pDC enables the expression of the cytotoxic molecules granzyme B and TRAIL, induces cDC1 maturation, and enhances the function of CD8^+^ T cells and NK cells in the TME, hence blocking tumor growth (183, 241). However, in some tumors, dysregulated and tolerated pDCs are always associated with poor prognosis of patients.

#### 2.2.7 Gamma Delta T cells

According to the T cell receptor classification, T cells can be mainly divided into the conventional αβT and Gamma Delta T cells(γδT). Interestingly, researchers have demonstrated that the γδT cell subsets that produce different cytokines have distinct metabolic requirements and play different roles in the growth of tumors. γδT cells, which produce IFN-γ, almost completely rely on glycolysis to adapt to the hypoxic environment in TME and have an antagonistic role (242). Currently, immunotherapy with PD-1/PD-L1 is commonly utilized in clinical practice and can effectively prolong the survival time of certain patients. Interestingly, recent studies have suggested that perhaps PD-L1-related immunoreactivity in bladder cancer may not require γδT cells and that IL-2 can promote the expression of CD122 in γδT cells to antagonize the tumor progression (243). Depending on the ability to recognize antigens without limitation of MHC-I, γδT cells have presented great potential in targeting therapy in colorectal cancer, melanoma-associated fibroblast, and glioblastoma (243–246). However, recently, researchers have revealed that γδT also plays a pivotal character in tumor growth and metastasis. The γδT cells which produce IL-17 play a crucial role in tumor development and metastasis. They increase mitochondrial mass and activity via oxidative metabolism, displaying selectively high lipid absorption and intracellular lipid storage (242, 247, 248). In addition, studies in recent years have reported that symbiotic bacteria in the lung can stimulate myeloid cells to produce Myd88-dependent IL-1β and IL-23, induce the activation of γδT cells that produce IL-17, and ultimately enhance tumor cell proliferation and inflammation (249).

**Figure 3:**
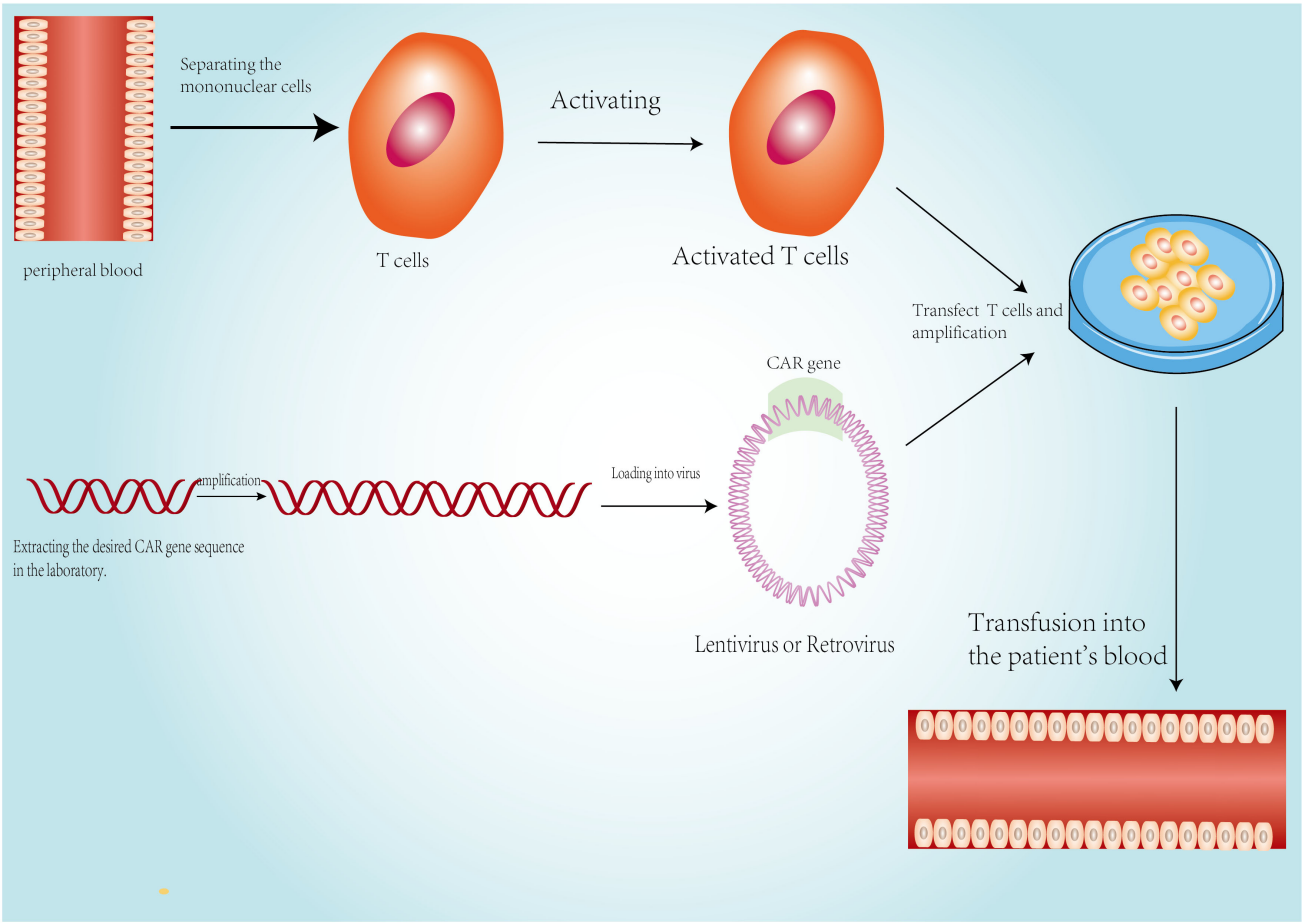
Schematic overview of CAR T-cell generation and infusion.

However, γδT cells can also be modified by CAR technology(**Figure 3**), and some studies have shown that the modified γδT cells have a stronger antagonistic ability against liver cancer (250). The inflammatory response is involved in every phase of tumor growth and progression.

## 3 Therapeutic strategies targeting the TME

Chemotherapy drugs have been considered harmful to the immune system in the past because of their bone marrow suppression. In gliomas, for example, temozolomide can decrease the absolute number of T cells in patients and increase the percentage of Treg cells in the immune system (251). Furthermore, in diffuse large B-cell lymphoma, the concentrations of MDSC and Treg in patients’ blood after chemotherapy were increased (252).

Recent perspectives on conventional chemotherapy, however, underscore an additional layer of complexity. As is shown in a recent study, despite the fact that millions of patients have been cured by these agents, the precise mechanisms underlying their therapeutic index remain only partially understood. Traditional models focused largely on direct cytotoxicity—through mechanisms such as mitochondrial apoptotic priming and DNA damage—but emerging evidence suggests that these cell‐autonomous effects might also intersect with immune modulation. This unresolved therapeutic index hints at a dual role for chemotherapy: while it can suppress immune function under certain conditions, it may also activate antitumor immunity by inducing immunogenic cell death and altering the tumor microenvironment (253).

In line with this, more and more evidence shows that chemotherapy can play a pivotal role in improving the anti-tumor immune response. In fact, it may help immunotherapy by activating the immune system instead of suppressing it (254). Acquired resistance to chemotherapy in gliomas is an important reason for the poor prognosis observed in these patients. For example, hypoxia in the tumor microenvironment (TME) upregulates HIF-1α, which promotes the conversion of macrophages to the M2 type; the subsequent secretion of vascular endothelial growth factor (VEGF) further enhances glioma progression and temozolomide resistance via the PI3K/Akt/Nrf2 pathway (255). Moreover, temozolomide treatment of glioma significantly induces the expression of CXCL1 and CXCL2, chemokines that promote the migration of bone marrow-derived inhibitory cells to tumors. Through paracrine secretion of S100A9, these chemokines activate Erk1/2 and p70S6K to further drive tumor progression. Blocking CXCL1/2 dramatically prolongs overall survival in mice treated with temozolomide, suggesting that these chemokines regulate tumor resistance during glioma treatment (256). In addition, temozolomide can induce the secretion of HMGB1 by tumor cells. HMGB1—a chromatin-binding protein—attracts various immune cells (257, 258) and plays a crucial role in anti-tumor immunity; notably, HMGB1 can induce M1-like polarization of tumor-associated macrophages (TAMs) and enhance glioma cell sensitivity to temozolomide (259). These observations underscore the potential for chemotherapy to synergize with immunotherapy. In line with this, integrative approaches to cancer immunotherapy emphasize the need to combine conventional chemotherapy with immunomodulatory strategies to overcome resistance mechanisms (260).

Furthermore, studies have shown that the dose of chemotherapeutics critically affects immunomodulation. For instance, standard doses (50 mg/kg×10 days) of temozolomide in gliomas markedly increase markers of T-cell depletion, such as LAG-3 and TIM-3, which are not observed with lower doses (25 mg/kg×10 days). In fact, the standard dose regimen abolished the survival advantage of PD-1 antibody therapy in syngeneic glioma models when combined with PD-1 blockade (261). Similarly, low-dose gemcitabine treatment not only increases the exposure of HMGB1 but also upregulates NKG2D ligands and activates NK cells, thereby improving patients’ anti-tumor immunity (262). These findings highlight the importance of optimizing chemotherapeutic dosing to maximize their immunostimulatory effects. Moreover, as advanced delivery technologies for cancer immunotherapy suggest, careful control over drug dosing and scheduling is essential to ensure that both chemotherapeutic and immunotherapeutic agents achieve effective concentrations at the tumor site (263).

Importantly, Chemotherapeutics can also stimulate the immune system through the combination of other drugs. For example, the combination of a CD47 blocker with temozolomide promotes tumor cell phagocytosis, which in turn enhances antigen cross-presentation and the activation of interferon-stimulated genes in antigen-presenting cells, leading to more efficient T-cell initiation (264). Similarly, when gemcitabine is used to treat nasopharyngeal carcinoma, it activates NF-κB to upregulate PD-1 in NK cells and PD-L1 in tumor cells; consequently, combining immunotherapeutic agents such as anti-PD-1 antibodies increases NK cell cytotoxicity by inhibiting the PD-L1/PD-1 checkpoint (265). Furthermore, gemcitabine combined with anti-PD-1 antibody immunotherapy exerts a stronger anti-tumor effect by enhancing the immune response mediated by Th1 lymphocytes and M1 macrophages (266). These combination strategies not only potentiate the immune-stimulating effects of chemotherapy but also provide a rationale for concurrently using immunotherapeutic agents to counteract chemotherapy-induced immunosuppression.

In parallel to these chemo‐immunomodulatory effects, a growing body of research on immunotherapies has highlighted their potential not only to directly activate anti‐tumor immunity but also to counteract the immunosuppressive tumor microenvironment. Notably, cancer immunotherapies – including immune checkpoint inhibitors and adoptive cell therapies – work by “releasing the brakes” on the immune system, thereby enhancing T-cell activity against tumor cells. However, as outlined in a recent review by Kennedy et al., these therapies also present a unique spectrum of toxicities that differ markedly from traditional chemotherapy. For instance, immune checkpoint inhibitors such as anti–CTLA-4, anti–PD-1, and anti–PD-L1 antibodies have been shown to restore cytotoxic T-cell function (216); yet, this reinvigoration of the immune response can inadvertently trigger immune-related adverse events (irAEs) affecting various organs including the skin, liver, gastrointestinal tract, and endocrine system. These adverse events, which range from mild rashes to severe conditions like hepatitis, colitis, and pneumonitis, require specific management strategies often involving immunosuppressive treatments such as corticosteroids (267). Consequently, when designing combination therapies, it is imperative to balance the benefits of enhanced anti-tumor immunity with the risk of immune-mediated toxicities.

Furthermore, innovative delivery technologies have emerged to enhance the efficacy and safety of immunotherapies (263). Advanced biomaterials—such as nanoparticles, implants, and scaffolds—are being engineered to enable targeted delivery of immunotherapeutic agents, thereby increasing accumulation in tumors, reducing off-target effects, and mitigating systemic toxicities (267). These platforms can protect the therapeutic cargo until it reaches the desired site and allow for controlled release in response to specific stimuli, such as pH changes or light exposure.

Current measures against tumor immunity include two main aspects: enhancing anti-tumor immunity and dampening pro-tumor immunity. The major strategies for enhancing anti-tumor immunity involve promoting the proliferation or activation of anti-tumor immune cells and synthesizing and releasing antitumor substances. For example, the DC vaccine is a promising immunotherapeutic approach, and related clinical trials are underway (97). As technology advances, therapies based on endogenous NK cells may become as routine as physical examinations for cancer prevention and treatment (9). Moreover, the use of TLR agonists, STING agonists, and antibodies targeting CD40 can further promote dendritic cell activation and function, thereby strengthening the body’s anti-tumor immunity (179, 268).

On the other hand, dampening pro-tumor immunity mainly involves reducing the production of immunosuppressive cells, inhibiting the release of suppressive cytokines, and promoting the conversion of immunosuppressive cells into anti-tumor effectors. Targeting MDSCs, Tregs, and immunosuppressive factors such as TGF-β, IL-10, and IL-35 can attenuate pro-tumorigenic effects (8). For instance, all-trans retinoic acid (ATRA) has been characterized as an inducer of MDSC differentiation into mature myeloid cells (88), while mast cell-derived metabolites like histamine can reverse the M2-like phenotype of TAMs toward an anti-tumor M1-like state (130).

Finally, recent advances in nanoparticle-based drug delivery have demonstrated that modifying traditional chemotherapeutic agents can also enhance immunotherapy. Nanoparticles not only improve the targeting of drugs to tumors with resistance but can also reduce the proportion of Treg cells in the TME and promote the depletion of MDSCs, thereby polarizing macrophages toward an M1 phenotype (269–272). Although these approaches remain largely in the preclinical stage, their promising prospects underline the potential of integrating chemotherapy with state-of-the-art immunotherapeutic strategies to achieve improved clinical outcomes (**Table 1**).

**Table 1:** List of the immune checkpoints and pharmacotherapy.

Cells	Immune checkpoints/ pharmacotherapy	Characteristics	Function	Ref
T cells	**HIF-1α**	inhibits CD8^+^T cell proliferation and cytotoxicity	Pro-tumor	(237)
	**Autophagosomes (TRAPs)**	inhibit T cell proliferation	Pro-tumor	(193, 203)
	**Adenosine**	affects T lymphocyte proliferation, initiation, and cytokine production	Pro-tumor	(119, 273–275)
	**CD47 blocker**	initiates more efficient T cell	Anti-tumor	(261)
	**MF-766**	promotes the infiltration of CD8^+^, CD45^+^, CD3^+^T cells	Anti-tumor	(177)
	**Transcription factors SOX4**	boost the TCR signaling pathway	Anti-tumor	(77)
	**Aryl hydrocarbon receptors (AHR)**	improves T cell function	Pro-tumor/Anti-tumor	(208, 209)
	**CTLA−4 (e.g., ipilimumab)**	An inhibitory receptor that competes with CD28 for binding to B7 (CD80/CD86) on antigen−presenting cells, thereby reducing IL−2 production and T−cell proliferation.	Pro−tumor	(268)
	**PARP11**	An intracellular enzyme induced in intratumoral CTLs that promotes downregulation of IFNAR1, leading to reduced type I interferon signaling and impaired CTL survival and cytotoxicity. Inhibition or genetic disruption restores CTL activity.	Pro−tumor	(64)
	**PD−1**	An inhibitory receptor on T cells; when engaged by its ligands PD−L1/PD−L2 (expressed on tumor or other cells), it induces T−cell exhaustion and decreases effector function, facilitating immune escape.	Pro−tumor	(65)
	**CCR8**	A chemokine receptor selectively overexpressed on tumor-infiltrating effector Tregs; it plays a key role in maintaining their immunosuppressive activity. Targeting CCR8 may selectively deplete Tregs from tumors and enhance anti−tumor responses.	Pro−tumor	(186)
	**CTLA−4**	Highly expressed on Tregs, CTLA−4 is critical for their suppressive function; it sequesters B7 molecules from APCs via transendocytosis, thereby limiting co-stimulatory signals to conventional T cells and reinforcing an immunosuppressive milieu.	Pro−tumor	(185)
NK cells	**Adenosine**	impairs the metabolic activities of NK cells	Pro-tumor	(118, 273–275)
	**Tumor-derived prostaglandin E2(PGE2)**	inhibit NK cells through EP_4_ dependent signals to decrease the secretion of cAMP and IFN-γ	Pro-tumor	(177, 276)
	**Transcription factors ID3**	promotes the growth of NK cells from bipotential NK/T progenitor cells in the thymus	Anti-tumor	(76)
	**Transcription factors SOX4**	regulates the formation of invariant natural killer T cells	Anti-tumor	(77)
	**NF-κB**	up-regulate PD-1 in NK cells and PD-L1 in tumor cells	Anti-tumor	(226, 262)
	**MF-766**	promotes the infiltration of NK cells	Anti-tumor	(177)
Macrophages	**HIF-1α**	promotes conversion of macrophages to M2 type	Pro-tumor	(236)
	**Adenosine**	inhibits macrophage activation	Pro-tumor	(118, 273–275)
	**Autophagosomes (TRAPs)**	induce M2 polarization	Pro-tumor	(202, 203)
	**Mast cell-derived metabolites (histamine)**	reverse the M2-like phenotype of TAMs to anti-tumor M1-like macrophages	Anti-tumor	(130)
	**MF-766**	induces the reprogramming of M1^-^like macrophages	Anti-tumor	(177)
	**NF-κB**	transcribe the M1 marker-encoding gene in M2 macrophages	Anti-tumor	(226, 264)
	**HMGB1**	induces M1-like polarization into the TAM and attracts various immune cells into TME	Anti-tumor	(256–258)
	**CAR-M1 macrophages**	1. phagocytize CAR-mediated malignancy 2. regulate antitumor immunity prevent the formation of solid tumors	Anti-tumor	(136)
	**Aryl hydrocarbon receptors (AHR)**	accelerates TAM recruitment and drives expression of the CD39 in TAM	Pro-tumor/Anti-tumor	(208, 209)
	**CSF1R signaling**	CSF1R, a receptor tyrosine kinase essential for macrophage survival and polarization, supports the differentiation of TAMs that secrete pro-angiogenic and growth factors, thereby promoting tumor progression and metastasis.	Pro−tumor	(185)
Neutrophils	**TGF-β**	is related to N2 polarization and INF-β or blocking TGF-β can stimulate TAN to polarize to N1	Pro-tumor	(145)
B cells	**Membrane B cell activator (BAFF)**	increase antibody production	Anti-tumor	(171)
Dendritic cells (DCs)	**HIF-1α**	enhances pDC recruitment into the tumor	Pro-tumor	(236)
	**Tumor-derived prostaglandin E2(PGE2)**	inhibits the IL-12 production of cDC1	Pro-tumor	(177)
	**MF-766**	promotes the infiltration of cDC	Anti-tumor	(135)
	**TLR agonists, STING agonists and CD40 antibodies**	promote DC activation and function	Anti-tumor	(184, 266)
Myeloid-derived suppressor cells (MDSCs)	**CXCL1/2**	promotes the migration of MDSC into tumors and ultimately interfere the infiltration of T cells in TME	Pro-tumor	(256)
	**PKR-like endoplasmic reticulum kinase (PERK)**	mediates the reprogramming of HSPC into a stereotypical MDSC precursor	Pro-tumor	(231)
	**CD36**	enhances fatty acid absorption and oxidation and induce the infiltration of MDSC in TME	Pro-tumor	(212)
	**MF-766**	reduces the MDSC in TME	Anti-tumor	(135)
	**All-trans retinoic acid (ATRA)**	induces of differentiation of MDSCs into mature myeloid cells	Anti-tumor	(97)
	**Interferon gene stimulator (STING)**	atagonize the expansion of MDSC	Anti-tumor	(233)

## 4 Conclusion

TME is variable, complex, and diversified and usually exhibits an immunosuppressive microenvironment. After immune cells enter the TME, a series of metabolic changes occur that often manifest as immune suppression and promotion of tumor development. Recent pan-cancer studies integrating single-cell and spatial transcriptomics have revealed that the TME’s heterogeneity extends far beyond cell type diversity; it is also defined by distinct spatial arrangements that evolve during tumor initiation, progression, and metastasis. For example, spatial transcriptomic analyses have identified specific stromal subpopulations—such as PGF^+^ tip endothelial cells—that are enriched in immune-depleted regions and are associated with epithelial-to-mesenchymal transition (EMT) and poor clinical outcomes (277). These studies demonstrate that spatial profiling technologies, including multiplex imaging and spatial indexing, can map gene and protein expression while preserving tissue architecture, thereby providing insights into the dynamic evolution of the TME at each tumor stage.

In addition, innovative approaches such as Perturb-map integrate CRISPR-based functional genomics with spatial transcriptomics to systematically dissect how specific gene perturbations affect the TME at single-cell resolution. Perturb-map studies have demonstrated that knocking out key regulators—such as the TGFβ receptor 2—leads to a fibro-mucinous remodeling of the TME and exclusion of T cells, thereby highlighting the critical role of extracellular gene functions in shaping tumor architecture (278). These findings illustrate that spatially resolved functional genomics can uncover not only cell-intrinsic mechanisms but also how intercellular communication and spatial organization contribute to immune evasion and therapeutic resistance.

Based on these characteristics, enhancing the role of immune cells in tumors by regulating the equilibrium between tumor metabolism and immune metabolism may open a new direction for tumor immunotherapy. Spatial analyses have further revealed that the metabolic reprogramming within the TME is closely linked to its spatial organization. In early tumor formation, for instance, interactions between cancer-associated fibroblasts (CAFs) and immune cells create metabolic niches that determine immune cell functionality. As tumors progress, regions with excessive extracellular matrix deposition and dysfunctional vasculature emerge, often harboring immune cells with altered metabolic states that contribute to therapy resistance (277, 279).

The treatment method for most tumors in the current era remains a combination of surgery, chemotherapy, and immunotherapy. However, the recurrence of cancer looms like a massive sword over the patient’s head, capable of striking at any time. Spatial profiling studies have shown that tumors often contain discrete niches—regions isolated from robust immune infiltration—that serve as reservoirs for residual malignant cells. These spatially confined microenvironments may enable tumor cells to evade therapy and later drive recurrence, emphasizing the need to understand and target these specific regions (280).

Moreover, despite numerous promising immunotherapy research outcomes, clinical applications still lag behind. For example, tumor vaccine therapy has demonstrated significant effects in preclinical studies over the past two decades, yet only a few such approaches have been successfully translated into clinical practice. Spatially resolved analyses indicate that the heterogeneous distribution of immune cells and their interactions with stromal components can profoundly influence therapeutic response. Consequently, integrating spatial profiling into clinical decision-making could refine immunotherapeutic strategies—such as tumor vaccines—by tailoring treatments to target specific microenvironmental niches, thereby improving the conversion efficiency from scientific research to clinical treatment (277, 279).

Collectively, these integrated spatial approaches—combining high-resolution imaging, spatial transcriptomics, and CRISPR-based functional genomics—provide a powerful toolkit for unraveling the intricacies of the TME. They not only offer mechanistic insights into how genetic alterations shape local immune landscapes but also pave the way for designing next-generation cancer therapies that precisely target the spatial and metabolic complexity of tumors.

In this schematic, the tumor mass is surrounded by various immune cell subsets whose phenotypes can either support or oppose tumor progression. CD8^+^ T cells (cytotoxic T lymphocytes) release IFN-γ and TNF-α, promoting tumor cell killing, but can become exhausted in nutrient-poor, hypoxic conditions. M1 macrophages secrete ROS and NO, mediating direct cytotoxicity and supporting Th1 responses. Conversely, M2 macrophages produce immunosuppressive factors such as IL-10 and VEGF, facilitating tumor growth, angiogenesis, and metastasis. B cells may present tumor antigens and produce tumor-specific antibodies, but certain regulatory B cells (Bregs) can secrete IL-10 and TGF-β, suppressing effector T cells. Tregs dampen immune responses via IL-10, TGF-β, and high-affinity IL-2 consumption. Neutrophils, similarly, can be polarized into an N1 subtype (with tumor-killing capacity) or an N2 subtype (promoting tumor progression through immunosuppression and angiogenesis). This dynamic interplay of immune cells underscores the complexity of the TME, where shifts in cytokine profiles and metabolic cues determine whether the immune system attacks or supports tumor cells.

This figure highlights how multiple immune-suppressive cells and molecules converge to inhibit T-cell–mediated antitumor responses. Tumor cells often upregulate PD-L1 to engage PD-1 on CD8^+^ T cells, dampening cytotoxic function. Myeloid-derived suppressor cells (MDSCs) release reactive oxygen species (ROS) and produce IL-10, further inhibiting T cells. Tumor-associated macrophages (TAMs), typically polarized to an M2-like phenotype, secrete IL-10 and TGF-β, which sustain immunosuppression. Regulatory T cells (Tregs) also release TGF-β and IL-10, hindering the activation of CD8^+^ T cells and natural killer (NK) cells. Meanwhile, dendritic cells (DCs) in the TME frequently have impaired maturation due to tumor-derived factors like PGE2, reducing their ability to present antigens effectively. Adenosine (ADO) produced in hypoxic regions binds to A2AR on T cells and NK cells, blunting antitumor activity. Collectively, these interactions establish an immunosuppressive milieu that allows tumor cells to evade immune surveillance.

This diagram illustrates the general process of creating chimeric antigen receptor (CAR) T cells for cancer therapy. First, peripheral blood is collected, and mononuclear cells are isolated. T cells within this fraction are activated and expanded *ex vivo*. Next, a lentiviral or retroviral vector carrying the CAR gene is introduced into the activated T cells, enabling them to express the tumor-specific CAR on their surface. These engineered CAR T cells are then further amplified in culture and ultimately transfused back into the patient. Once reinfused, CAR T cells recognize and kill tumor cells via the newly expressed CAR, offering a potent, antigen-directed immunotherapy for both hematologic and, in emerging studies, certain solid tumors.
